# Serum Homocysteine Level in Parkinson's Disease and Its Association with Duration, Cardinal Manifestation, and Severity of Disease

**DOI:** 10.1155/2018/5813084

**Published:** 2018-05-02

**Authors:** Payam Saadat, Alijan Ahmadi Ahangar, Seyed Ehsan Samaei, Alireza Firozjaie, Fatemeh Abbaspour, Sorrayya Khafri, Azam Khoddami

**Affiliations:** ^1^Mobility Impairment Research Center, Health Research Institute, Babol University of Medical Sciences, Babol, Iran; ^2^Social Determinants of Health Research Center, Health Research Institute, Babol University of Medical Sciences, Babol, Iran; ^3^Cellular and Molecular Biology Research Center, Health Research Institute, Babol University of Medical Sciences, Babol, Iran; ^4^Clinical Research Development Center, Ayatollah Rohani Hospital, Babol University of Medical Sciences, Babol, Iran; ^5^Department of Statistic and Epidmiology, School of Medicine, Babol University of Medical Sciences, Babol, Iran

## Abstract

**Background and Purpose:**

Due to the high prevalence of Parkinson's disease (PD) in the elderly, a large financial burden is imposed on the families and health systems of countries in addition to the problems related to the mobility impairment caused by the disease for the patients. Studies on controversial issues in this disease are taken into consideration, and one of these cases is the role of serum homocysteine level in Parkinson's patients. In this study, the serum level of homocysteine and its association with various variables in relation to this disease was compared with healthy individuals.

**Materials and Methods:**

In this study, 100 patients with PD and 100 healthy individuals as control group were investigated. Serum homocysteine level and demographic and clinical data were included in the checklist. Data were analyzed by SPSS version 23. In all tests, the significance level was below 0.05.

**Results:**

The mean level of serum homocysteine in case and control groups was 14.93 ± 8.30 and 11.52 ± 2.86 *µ*mol/L, respectively (95% CI: 1.68; 5.14, *P* < 0.001). In total patients, 85 had normal serum homocysteine level, while 15 had high serum homocysteine level. In controls, the homocysteine level was 98 and 2, respectively (*P*=0.002). In multivariate logistic regression analysis, serum homocysteine level higher than 20 *µ*mol/L was accompanied by 8.64-fold in Parkinson's disease involvement (95% CI: 1.92; 38.90, *P*=0.005).

**Conclusion:**

Increasing serum homocysteine level elevates the rate to having PD. Serum homocysteine levels did not have any relationship with the duration of the disease, type of cardinal manifestation, and the severity of Parkinson's disease.

## 1. Introduction

PD is the second progressive neurodegenerative disease and one of the important and common causes of mobility impairment in the elderly that occurs in all races with almost equal gender distribution [1]. Its prevalence is increased with increasing age [2]. This disease often occurs in individuals older than 60 years and is sporadic in most cases, but the incidence of disease in families is also observed in about 15% of cases; genetic and environmental factors are involved in the etiology of the disease [3, 4]. Agricultural pesticides [5] are one of the environmental factors that affect the disease. On the other hand, cigarette [6] and caffeine [7] seem to have a protective effect. Homocysteine is produced by the metabolism of the methionine amino acid, and there is a possibility of its reuptake by remethylation and converts into methionine. The enzyme that performs the recent reaction requires folate and B12. This enzyme also controls the serum level of homocysteine by coenzymes B12, folate, B6, and choline. Hence, the deficiency of these vitamins leads to the accumulation of homocysteine [8]. The deficiency of folate and B12 leads to atrophy of the neurons of CA1 region of the hippocampus and disruption of cognitive processes with increasing homocysteine [9].

Increasing plasma homocysteine level is associated with elevating the risk of systemic vascular disease through enhancement of the adverse effects of risk factors such as hypertension, smoking, and lipid and lipoprotein metabolism, as well as acceleration of development of inflammation. Increased homocysteine also has been reported during recent decades in neurological diseases without vascular origin such as Alzheimer's disease [10] and idiopathic Parkinson's disease [11, 12].

Furthermore, in the research conducted on PD patients, homocysteine level of cerebrospinal fluid has been reported higher than normal [11]. Homocysteine leads to damage to MPTP-dependent (1-methyl,4-phenyl-1,2,5, and 6 tetrahydropyridine) dopaminergic cells [13].

On the other hand, according to some researchers, high homocysteine level in PD patients may be due to dopamine compound treatment [14]. However, with regard to homocysteine and Parkinson's disease, the available findings are controversial. Due to the high prevalence of PD in the elderly, a large financial burden is imposed on the families and health systems of countries in addition to the problems related to the mobility impairment caused by the disease for these patients. Hence, conducting research projects on different aspects of this disease can be important. Given these issues, there is a controversy about the relationship between homocysteine and PD. Even if there is this relationship, there is vagueness regarding the association between homocysteine with some variables of Parkinson's disease.

Variables such as demographic characteristics of patients and issues such as type of occupation or patients' degree of education may have an impact in the serum level of homocysteine. To answer these questions, this study was designed and carried out. In the case of the existence of relationships between homocysteine and PD and specifically with some of its related variables in this study and its confirmation with other studies, it may be possible to use these results in preventing or treating this disease.

## 2. Materials and Methods

This cross-sectional and case-control study was conducted at the Neurology Clinic of Ayatollah Rouhani Hospital in Babol during 2015-2016. The proposal of this study was approved by the Research Council of Babol University of Medical Sciences (number 3183) and the Ethics Committee of Research Studies of Babol University of Medical Sciences. One hundred new PD patients who have not received treatment yet were selected as the case group and 100 healthy individuals as the control group. Two groups of case and control were matched according to age and gender.

Inclusion criteria were the definite diagnosis of PD based on the Movement Disorder Society (MDS) Clinical Diagnostic Criteria for Parkinson's Disease [15], with four major cardinal manifestations including tremor, hypokinesia, rigidity, and postural instability [16], under the supervision of a neurologist who is responsible for the study.

Grading of motor impairment was done based on the Movement Disorder Society- (MDS-) sponsored revision of the Unified Parkinson's Disease Rating Scale (MDS-UPDRS) [17]. Scoring severity of disease was done based on modified Hoehn and Yahr staging; thus, the severity of the disease was classified into three classes of 1-2 (mild), 2.5–3 (moderate), and more than 3 (severe) [18].

Demographic information (age, gender, occupation, level of education, and economic status) of PD cases and control groups and clinical status of PD patients (duration, cardinal manifestation, and severity of disease) were determined.

Exclusion criteria were as follows: patients with Parkinson's disease who have already been receiving medical treatment, including levodopa, disease caused by the use of certain neuroleptic drugs or toxins, Parkinsonism associated with other neurologic diseases, and Parkinson's disease patients with renal hepatic failure, pharmaceutical supplement consumption, and patient's dissatisfaction to participate in the study.

Exclusion criteria for control group were individuals having any symptoms or signs of Parkinson's syndrome, renal and hepatic failure, and pharmaceutical supplement consumption. The ages of patients were classified into three classes: under 60, 60–80, and older than 80 years.

In terms of education, patients were divided into two classes: illiterate and literate. Based on type of job, they were classified as an employee, a freelancer (mostly farmers), and an unemployed (including housewives). Duration of the disease was classified into three classes: less than 5 years, between 5 and 10 years, and more than 10 years. For ruling out other diagnoses, brain imaging was performed for the patients. Routine laboratory tests and measurements of serum homocysteine level were done in the laboratory of Ayatollah Rouhani Hospital in Babol. In both the case and control groups, serum homocysteine level was measured by the ELISA method [19]. Heparin plasma samples were centrifuged within 30 minutes in the laboratory to prevent its false increase due to the release of homocysteine from red blood cells. Separation and freezing the samples after collection was done within an hour. Serum homocysteine levels less than 12 *μ*mol/L were desirable, and levels higher than 15 *μ*mol/L were considered at risk of vascular disease [20]. We consider serum homocysteine levels higher than 20 *μ*mol/L associated with an increased risk of PD, and based on this, the serum homocysteine level was classified into two classes: normal (20 *μ*mol/L and less) and high (more than 20 *μ*mol/L).

In addition to the results of serum homocysteine level, demographic information (age, gender, occupation, and level of education) of the case and control groups and clinical status (duration, cardinal manifestation, and severity of disease) of the patient group were entered in the study checklist.

The information and laboratory results obtained from the patients and the control group were entered into SPSS version 23 software and analyzed using the statistical independent *t*-test and one-way ANOVA. They were also investigated using multivariate logistic regression test by modifying the interfering variables that have a role homocysteine level in Parkinson's disease. In all tests, the significance level was less than 0.05.

## 3. Results

The mean age of 100 PD cases was 69.72 ± 9.88 years and the control group was 69.37 ± 9.88 years (the age range was 42–97 years in both groups). Male and female in the case group were 53 and 47 years, respectively, and both were 50 years in the control group (Table 1). In terms of age, the patients were as follows: 16 cases were below 60 years, 72 cases were between 60 and 80 years, and 12 individuals were older than 80 years. In relation to the level of literacy, 61 individuals were illiterate and 39 individuals were literate. There was no significant difference between the case and control groups in any of these variables (Table 1).

According to the duration of the disease, 65 individuals involved were less than 5 years ago, 27 were between 5 and 10 years ago, and 8 were more than 10 years ago. In terms of cardinal manifestation of patients, 62 individuals suffer tremor, 13 had rigidity, 22 with bradykinesia, and 3 had postural instability. The severity of the disease of the patients was 1-2 (35 individuals), 2.5-3 (44 individuals), and more than 3 (21 individuals) (Table 2).

Accordingly, 85 patients were with normal serum homocysteine level and 15 patients were with increased serum homocysteine level in the case group. This number was 98 and 2 patients, respectively, in the control group. Accordingly, there was a significant difference between the two groups (*P*=0.002). The mean of serum homocysteine level was 8.30 ± 14.93 *μ*mol/L in the case group and 2.86 ± 11.52 *μ*mol/L in the control group (Table 3), and there was a significant difference between the two groups in this regard (*P* < 0.001; 95% CI: 1.68; 514). In the Pearson correlation analysis, the correlation of homocysteine level with the control group and case group was −0.02 and 0.148, respectively, but none of them was statistically significant (*P* was 0.840 and 0.140, resp.). There was a significant difference in the patient group compared to the control group, male and female, between the age of 60 and 80 years, and in the literate and illiterate individuals in terms of the mean of the serum homocysteine level (*P* was 0.005, 0.009, 0.001, 0.047, and 0.001, resp.) (Table 3).

The serum homocysteine level has increased with the increasing severity of the disease, but this increase was not statistically significant. At the severity of 2.5–3 of Parkinson's disease compared to other severities, the higher frequency of patients with increased serum homocysteine was observed (8 patients). Nonetheless, this difference was not significant between the three different classes of the severity of Parkinson's disease (Table 4). Homocysteine level in patients with Parkinson's disease increased with increasing duration of the disease, but this difference was not significant between patient groups (Table 4). The serum homocysteine level in patients with Parkinson's disease was not significant in this study according to their cardinal manifestation (Table 4).

The mean level of homocysteine of employed people was 5.39 ± 13.09 *μ*mol/L, freelancer (farmers) was 7.29 ± 14.79 *μ*mol/L, and unemployed (housewives) was 9.80 ± 15.85 *μ*mol/L. Accordingly, the mean level of homocysteine of the farmers and unemployed/housewives was higher than the employed people, but this difference was not statistically significant (Table 5).

The amount of risk of independent factors for Parkinson's disease was investigated using logistic regression analysis by modifying the involved factors such as age and gender. Accordingly, serum homocysteine levels higher than 20 *μ*mol/L increased 8.64-fold the chance of having PD (*P*=0.005, 95% CI: 1.92; 38.90) (Table 6).

## 4. Discussion

According to this study, the serum homocysteine level in PD patients was significantly higher than the healthy subjects. Although many factors can influence this level difference, all of which may not be considered in our study, it can be said that the risk of getting involved in Parkinson's disease was estimated to be 8.64-fold with increasing the serum homocysteine level more than 20 *μ*mol/L based on logistic regression multivariate analysis.

One of the most important factors that can affect the serum homocysteine level is the type of diet. Regarding the available data, the type of diet can play an important role in Parkinson's disease; for example, vitamin B12 and folate, whose amount varies in different diets, are involved in controlling serum homocysteine levels [8].

Definitely, how high serum homocysteine level can increase the risk of Parkinson's disease is not clear, and it is even possible that the high serum homocysteine level may be due to the effect of disease on patients rather than that causes PD. In some previous studies, increasing homocysteine level in the general population was associated with the risk of mild cognitive impairment, atherosclerosis, and neurodegenerative diseases such as Alzheimer's disease, vascular dementia, and Parkinson's disease [21–24].

Although the empirical observations confirm this issue, many studies do not confirm this relationship and the role of homocysteine in the pathogenesis of Parkinson's disease is currently unknown. In a study conducted by Rodriguez-Oroz et al. in 2009, the findings showed an increase in serum homocysteine in patients with Parkinson's disease compared to healthy subjects, as in our study [21].

In a study by Song et al. in 2016, the serum homocysteine level in patients with PD was higher than that in healthy subjects [22], which is consistent with our study. They also investigated the effect of levodopa on increasing serum homocysteine in patients, and according to their findings, the patients with levodopa treatment had higher levels of homocysteine than others. In another study conducted by Hu et al. in 2013, findings showed no significant difference in homocysteine level in the untreated patients compared to healthy subjects [14].

According to the findings of our study, there was no significant difference between the male and female PD patients in terms of the mean of serum level of homocysteine, although there was a significant difference between the two groups of patients and healthy subjects in terms of gender. Based on the study of Kocer et al. in 2016, there was a significant difference between the two groups of patients in terms of gender [25]. They report higher serum homocysteine level in men than women, which was not consistent with our study as mentioned; there was no significant difference between serum homocysteine levels in the two genders of the patient group, and our findings were not consistent with these studies. In this regard, we cannot provide an acceptable justification for this inconsistency, but perhaps the small number of patients in our study had led to the lack of significance of the serum homocysteine level in these two groups. Certainly, as mentioned earlier, there was a significant difference between the two groups of patients and controls in terms of men and women and their serum homocysteine level. In other words, the grade of difference of homocysteine serum level in the patient groups in comparison with the control group was higher in women than men.

The mean of serum homocysteine level was not significant based on the degree of education of patients, and also difference between different ages was not significant. In this study, the mean of serum homocysteine level increased in patients with increasing severity of PD. Although this difference was not statistically significant, there was no significant relationship of the increased homocysteine level of patients in terms of duration of the disease in the study of Kocer et al. [9], which was consistent with our study.

Based on the results of this study, there was not a significant difference in terms of the relationship between the duration of involvement of Parkinson's disease patients and their serum homocysteine level; perhaps, the reason is that they did not receive any medical treatment (dopamine compounds). And this hypothesis justifies that the high serum homocysteine level in Parkinson's disease may be due to receiving dopaminergic drugs. In another study conducted by Religa et al. in 2006, serum homocysteine level of patients with Parkinson's disease was higher than healthy subjects; moreover, there was a significant relationship between the duration of the disease and increased serum homocysteine [26]. However, the results of this study were not consistent with the results of our study.

Among other variables studied in this study, serum homocysteine level was based on the type of their employment; accordingly, there was no significant difference between the groups of patients and control and different variables studied in patients with Parkinson's disease. In the previous studies conducted, the effect of different occupations on Parkinson's disease had been due to the effects of occupational factors such as agricultural pesticides [5] or industrial toxins such as metals, solvents, and organophosphate compounds on this disease [27], and the results of this study also show that the effect of the type of occupation on PD is not due to its direct effect on serum homocysteine level.

There was not a significant difference between changes in serum homocysteine levels in patients with Parkinson's disease in terms of the type of their cardinal manifestation. The results of the few studies that were conducted and searched were also similar to the results of our study [23, 25]. One of the interesting aspects in Parkinson's disease is its association with the digestive tract. Intestinal dysfunction occurs in most patients before the onset of motor manifestations of the disease; it can be indicative of a possible contribution of intestinal dysfunction in the pathogenesis of PD.

High *α*-synuclein protein that is present in the intestines of PD patients increases intestinal permeability, resulting in synucleinopathy in the intestines of these individuals; this happens before the occurrence of cardinal manifestation in PD patients. Regarding these and many other findings, the association between the digestive tract and Parkinson's disease (the gut axis in PD patients) has been very much considered in the recent study [28].

The strengths of this study were selection of patients with Parkinson's disease who were not under any medical treatment so far and investigation of changes in the serum homocysteine level in patients with Parkinson's disease due to the type of their cardinal manifestation and some other variables, although the small number of cases and control groups studied was the limitation of this study.

Our small sample size might have led to loss of the power statistical analysis. To determine more precisely the relationship between homocysteine and PD, the study of measurement of serum homocysteine level in patients with Parkinson's diagnosis with more cases, and more healthy controls, and also the study on the patient group with Parkinson's disease diagnosis with and without dopamine compounds treatment are recommended.

## 5. Conclusion

According to this study, an increase in serum homocysteine level in PD patients has been observed, which can be one of the factors associated with to the disease. High age was one of the factors associated with increased serum homocysteine level in patients. There was no relationship between the severity of the disease and the serum homocysteine level. The mean of serum homocysteine level increased in patients with increasing severity of Parkinson's disease.

There was not a significant difference in terms of the relationship between the duration of involvement in Parkinson's disease patients with their serum homocysteine level. Moreover, there was no significant relationship between serum homocysteine level of patients in terms of duration of the disease. There is no exact mechanism for increasing serum homocysteine level in Parkinson's disease patients, and more studies must be conducted to clarify this issue. By clearing up these issues, measuring serum homocysteine level can be suggested in the laboratory screening test to identify the patients at risk for Parkinson's disease. In the future, modification of serum homocysteine level may play a role in preventive methods or treatment of Parkinson's disease.

## Figures and Tables

**Table 1:** Demographic characteristic of Parkinson's patients and control group.

Variables	Case (%) (*n*=100)	Control (%) (*n*=100)	*P* value
*Gender*			
Male	53 (53%)	50 (50%)	0.777
Female	47 (47%)	50 (50%)	
*Age (year)*			
<60	16 (16%)	16 (16%)	1.00
60–80	72 (72%)	72 (72%)	
>80	12 (12%)	12 (12%)	
*Education*			
Literate	39 (39%)	31 (31%)	0.299
Illiterate	69 (69%)	61 (61%)	

**Table 2:** Clinical features of Parkinson's disease.

Features	Patients, *n* (%)
*Duration of the disease*	
Less than 5 years	65 (65%)
5–10 years	27 (27%)
More than 10 years	8 (8%)
*Cardinal manifestation of the disease*	
Tremor	62 (62%)
Rigidity	13 (13%)
Bradykinesia	22 (22%)
Postural instability	3 (3%)
*Severity of deterioration of the disease (based on modified Hoehn and Yahr staging)*	
1-2 (mild)	35 (13%)
2.5-3 (moderate)	44 (44%)
More than 3 (severe)	21 (21%)

**Table 3:** (Mean) serum homocysteine level of the case and control groups (*μ*mol/L).

Demographic pattern	(Mean) serum homocysteine level		*P* value
	Case (*n*=100)	Control (*n*=100)	
*Gender*			
Male	14.73 ± 6.46	11.87 ± 2.72	0.005
Female	15.17 ± 10.05	11.18 ± 10.05	0.009
*Age (years)*			
<60	12.30 ± 3.21	12.01 ± 0.52	0.776
60–80	15.54 ± 8.20	11.78 ± 2.87	0.001
>80	14.79 ± 6.02	11.18 ± 2.15	0.063
*Education*			
Literate	14.19 ± 8.51	10.96 ± 2.79	0.047
Illiterate	15.41 ± 8.20	11.78 ± 2.87	0.001

**Table 4:** Frequency of different clinical features of Parkinson's disease based on serum levels of homocysteine.

Serum homocysteine level (*μ*mol/L)	Parkinson's disease features				*P* value
	Duration of the disease (years)				
	≤5	5–10	>10		
≤20	57 (87/7%)	21 (77/8%)	7 (87/5%)		0.572
>20	8 (12.3%)	6 (22.2%)	11 (12.5%)		

	Cardinal manifestation of the disease				
	Postural instability	Tremor	Rigidity	Bradykinesia	
≤20	2 (66.7%)	53 (85.5%)	11 (84/6%)	19 (86.4%)	0.841
>20	1 (33.3%)	9(14.5%)	2 (15.4%)	3 (13.6%)	

	Severity of the disease based on modified Hoehn and Yahr staging				
	1-2 (mild)	2.5-3 (moderate)	>3 (severe)		
≤20	33 (94.3%)	36 (81.8%)	16 (76.2%)		0.154
>20	2 (5.7 %)	8 (18.2%)	5 (23.8%)		

**Table 5:** The mean serum homocysteine level by occupation in the patients group.

Variables	Occupation			*P* value
	Employed (*n*=22)	Freelancer (mostly farmers) (*n*=29)	Unemployed/housewives (*n*=49)	
Means of homocysteine	13.09 ± 5.39	14.79 ± 7.29	15.85 ± 80	0.433

**Table 6:** Logistic regression multivariate analysis to determine the role of independent risk factors in Parkinson's disease.

Variables		OR	95% CI	*P* value
Serum homocysteine levels higher than 20		8.64	1.92–38.90	0.005
Severity of illness	1-2	Ref.	Ref.	0.172
	2.5-3	3.67	0.72–18.53	0.116
	>3	5.16	0.9–29.53	0.065
